# Association of Anthropometric and Lifestyle Parameters with Fitness Levels in Greek Schoolchildren: Results from the EYZHN Program

**DOI:** 10.3389/fnut.2018.00010

**Published:** 2018-02-09

**Authors:** Giannis Arnaoutis, Michael Georgoulis, Glykeria Psarra, Anna Milkonidou, Demosthenes B. Panagiotakos, Dafni Kyriakou, Elena Bellou, Konstantinos D. Tambalis, Labros S. Sidossis

**Affiliations:** ^1^Department of Nutrition and Dietetics, School of Health Science and Education, Harokopio University, Athens, Greece; ^2^Department of Kinesiology and Health, Division of Life Sciences, School of Arts and Sciences, Rutgers University, The State University of New Jersey, New Brunswick, NJ, United States

**Keywords:** physical inactivity, obesity, children, Mediterranean diet, fitness tests, central obesity

## Abstract

**Objective:**

The aim of the study was to evaluate physical fitness (PF) and identify its anthropometric and lifestyle determinants in a sample of Greek schoolchildren.

**Methods:**

The study sample consisted of 335,810 schoolchildren (♂: 51.3%, 6–18 years old). Students’ anthropometric parameters and PF levels—assessed *via* the Eurofit test battery—were measured by trained physical education teachers and evaluated according to the available norms, while their lifestyle habits were assessed through a questionnaire.

**Results:**

In all applied PF tests, students’ performance was negatively associated with the presence of obesity and central obesity, defined through international criteria for body mass index and waist to height ratio, respectively. According to multiple logistic regression analysis, the presence of overweight/obesity [odds ratio (OR): 4.43, 95% confidence interval (CI): 3.98–4.93], low adherence to the MD (KIDMED ≤ 3) (OR: 1.27, 95% CI: 1.09–1.48), and increased time spent in sedentary activities (>2 h per day) (OR: 1.16, 95% CI: 1.03–1.29) were positively associated with poor PF, after adjusting for age and sex. In contrast, for every 1 day increase in the weekly frequency of engagement in athletic activity, the probability of poor PF decreased by 26% (OR: 0.74, 95% CI: 0.72–0.77). In a similar model, the presence of central obesity emerged as an even stronger possible predictor of poor PF (OR: 5.20, 95% CI: 4.66–5.78), compared to the presence of general obesity.

**Conclusion:**

Higher general or abdominal adiposity, as well as the adoption of a low-quality diet and a sedentary lifestyle, is strongly associated with low PF levels during childhood.

## Introduction

Physical fitness (PF) is defined as “the ability to carry out daily tasks with vigor and alertness, without undue fatigue and with ample energy to enjoy leisure-time pursuits and to meet unforeseen emergencies” (1). PF is a complex term involving cardiorespiratory endurance, muscle endurance, and muscle strength, as well as flexibility, balance, agility, and coordination. Although PF is the outcome of a multifactorial behavior, recent data explain differences between subjects, in part, by genetic variation. More precisely, specific genetic loci have been found to relate performance and health-related fitness phenotypes. Polymorphisms in several genes have been found to be associated with cardiorespiratory fitness, including β2-adrenergic receptor (ADRB1) and β2-adrenergic receptor (ADRB2), and baseline muscle strength, including vitamin D receptor (VDR) (2–4). Therefore, PF actually represents a set of attributes or abilities that an individual has or achieves in order to perform physical activity efficiently. A high PF level in childhood is considered essential for the maintenance of good health and general wellness. According to accumulating epidemiologic evidence, high PF level is related to a favorable body composition, improved skeletal health, protection against cardiometabolic risk factors (e.g., hypertension and dyslipidemia), as well as improved mood, psychological health, academic performance, and quality of life (5). In addition, longitudinal studies reveal a significant graded association between low PF levels in adolescence and an increase in the risk of cardiovascular disease and early death in adulthood (6, 7). However, worldwide data indicate significantly low percentages of children who are classified as physically fit. A recent systematic review which included data from 50 countries indicated extremely low levels of fitness for children and youth aged 9–17 years, especially in the South America and South European countries (8). In Greece, Tambalis and his colleagues (9) have also demonstrated a significant increase in the percentage of children classified in the lowest quartile of aerobic performance from 21 to 48.2% in girls and from 25.7 to 38.7% in boys, during an 11-year period (1997–2007). Thus, promoting PF in childhood, through identifying and intervening on its modifiable determinants, is crucial for public health strategies aiming at promoting the health of the pediatric population.

Several factors associated with physique and lifestyle can potentially have an impact on the PF levels in childhood; however, their correlation has not been systematically investigated in a large number of large-scale studies so far. Regarding anthropometric parameters, longitudinal inverse trends in obesity and PF levels have been reported in schoolchildren (9), a fact that raises concerns given the global obesity epidemic. Indeed, childhood obesity and abdominal adiposity are considered as escalating health problems globally (10–13), and policy interventions for their management are urgently needed. Moreover, since they persist through adulthood (14) and pose a great threat for children’s health being associated with increased cardiovascular disease risk, as well as endocrine, gastrointestinal, respiratory, musculoskeletal, and psychological disorders (15–17). However, reductions in children’s PF levels have been reported to be independent of the increasing trends in obesity (9), suggesting that other factors, probably related to lifestyle, are also significant determinants of PF levels in the youth. The increase in the prevalence of pediatric obesity and associated comorbidities has been mostly attributed to environmental factors and specifically poor lifestyle habits, as documented in several studies both in developed and developing countries (18). For instance, the “nutrition transition phenomenon” has gradually led to the westernization of children’s diet and a gradual shift from traditional prudent dietary patterns, including the Mediterranean diet (MD), to patterns characterized by increased consumption of fast food, sweets, soda, and meat products (19–21). Moreover, physical inactivity is nowadays estimated to be common among the youth, as indicated by epidemiological data which show that only a small proportion of children and adolescents achieve the recommended levels of physical activity for health promotion [~25% of children (6–15 years old) are classified as at least moderately active for 60 min per day on at least 5 days per week] (22–24).

Considering the importance of a high PF level in childhood, as well as the lack of studies assessing its correlation with lifestyle in youth, the purpose of the present study was to determine PF levels, to explore the association between the performance in various PF tests and anthropometric and lifestyle parameters, as well as to identify the most significant modifiable predictors of total poor PF level in a nationally representative sample of Greek schoolchildren.

## Materials and Methods

### Participants

Population-based data were derived from a nationwide school-based survey, i.e., the EYZHN (National Action for Children’s Health) program, performed under the auspices of Harokopio University of Athens and the Greek Ministry of Education, Research and Religious Affairs and aiming to record health- and lifestyle-related parameters of the total student population of Greece. In the context of the EYZHN program, all Primary and Secondary schools of Greece (both public and private and from all geographical districts of the country) were invited to participate, following a formal letter sent from the Greek Ministry of Education, Research and Religious Affairs to all Primary and Secondary Education Boards of the country. For the current work, anthropometric, dietary, physical activity, and PF data, along with information on age and sex, from a total sample of 335,810, 6–18 year old students, who participated in the EYZHN program during the school year 2014–2015, were analyzed. Data were collected from March 2015 to May 2015, and participation rate was 40% of the total Greek student population. All students had Greek nationality, were informed about the study experimental procedures, and a verbal assent was obtained from each participating child, along with a written informed consent provided by their parents. The study was conducted according to the guidelines laid down in the Declaration of Helsinki, and all procedures involving human subjects/patients were approved by the Review Board of the Ministry of Education and the Ethical Committee of Harokopio University of Athens.

### Anthropometric Measurements

All anthropometric measurements were performed by trained physical education professionals at schools, and a standardized procedure was implemented, along with a systematic calibration of the devices (e.g., weight scales), in order to ensure maximum validity and accuracy. Students’ body mass and stature were measured in the morning, without shoes using a standardized protocol (25). Body mass was measured in the standing upright position with electronic scales with a precision of 100 g. Stature was measured to the nearest 0.1 cm with the children’s weight equally distributed on their feet and their head, back, and buttocks on the vertical line of the height gage. Children body mass index (BMI) was calculated, as the ratio of the body weight to the square of body height (kg/m^2^) based on the aforementioned measurements. BMI values were transformed into BMI *z* scores using WHO reference values for pediatric BMI (26). The presence of overweight and obesity was defined according to the international age- and gender-specific criteria proposed by Cole et al. (27). For the purpose of the present analysis, underweight children were incorporated with their normal weight counterparts. Waist circumference (WC) was measured to the nearest 0.1 cm midway between the lowest rib and the superior border of the iliac crest at the end of normal expiration with the use of a non-elastic measuring tape positioned at a level parallel to the floor and with the subject in a standing position. The evaluation of abdominal adiposity was based on waist to height ratio (WHtR), calculated as the ratio of WC (cm) to height (cm). Central obesity presence was defined as WHtR ≥ 0.5, given that this specific cutoff point has been found suitable for the estimation of abdominal adiposity in children and for the prediction of cardiometabolic abnormalities (28, 29).

### Assessment of PF Levels

The Eurofit PF test battery was used to evaluate children PF levels (30), initially proposed by the Council of Europe and used systematically from many European countries during the last few decades. The battery consists of five tests, i.e., (a) a multistage 20 m shuttle run test (20 m SRT), to estimate cardiorespiratory endurance; (b) a maximum 10 × 5 m shuttle run test (10 × 5 m SRT) from a standing start to evaluate speed and agility; (c) a sit-ups (SUs) test in 30 s, in which the student lies on the mat with the knees bent at right angles, with the feet flat on the floor and held down by a partner, to measure the muscular endurance of the abdominals and hip flexors; (d) a standing long jump (SLJ), where the children are asked to bend their knees with their arms in front of them, parallel to the ground, then swing both arms, push off vigorously and jump as far as possible, trying to land with their feet together and stay upright, to evaluate lower body muscular strength; and (e) a sit and reach (SR) test that involves sitting on the floor with legs stretched out straight ahead without shoes to measure flexibility. In detail, the 20 m SRT test consists of measuring the number of laps completed by participants running up and down between two lines in groups, set 20 m apart, at an initial speed of 8.5 km/h which increases by 0.5 km/h every minute, using a pre-recorded audio tape (31, 32). The test is terminated when participants stop due to fatigue or when they fail to reach the end line concurrent with the signals on two consecutive occasions, and the last completed stage or half-stage is recorded. For the SR test, the soles of the feet are placed flat against a box. With the palms facing downwards, and the hands on top of each other or side by side, the participant reaches forward along the measuring line as far as possible. The score is recorded to the nearest centimeter as the steady distance reached by the hand for at least 2 s, using 15 cm at the level of the feet. Two trials were allowed for the SLJ, SR, SUs, and 10 × 5 m SRT, with the best performance of each recorded. All five fitness tests were administered during the physical education class by physical education professionals, who were instructed through a detailed manual of operations and followed a standardized procedure of measurements in order to minimize the inter-rate variability among schools.

Student performance in PF tests was evaluated based on the recently published PF normative age- and sex-specific values for 6- to 18-year-old Greek boys and girls (33). Specifically, for each of the five PF tests applied, a performance ≤25th percentile was considered as low, between the 25th and 75th as average, and ≥75th as high. Based on this categorical classification, a binary outcome (0 versus 1) of low PF was utilized for each PF test applied. For example, low PF in the 20 m SRT was defined as a performance ≤25th percentile of normative age- and sex-specific values of the 20 m SRT. Using the abovementioned binary classification for each PF test, a combined variable representing total poor PF (0 versus 1) was constructed. This variable was defined as the combined low performance (≤25th percentile) in all five PF tests applied. Therefore, student total PF was characterized as poor if performance was ≤25th percentile of normative age- and sex-specific values in all five PF tests applied.

### Lifestyle Assessment

Participating children’s lifestyle (dietary and physical activity habits) habits were recorded *via* the use of an electronic questionnaire that was completed at school with the presence and assistance of their teachers and/or Information Technology professors, all previously provided with specific written guidelines for its proper completion, in order to provide an accurate reflection of their habits and for a standardized evaluation protocol to be implemented among all participating schools. Regarding students’ dietary habits, these were assessed through the KIDMED (Mediterranean Diet Quality Index for children and adolescents), developed by Serra-Majem et al. (34). The KIDMED index was developed in an attempt to combine the MD guidelines for adults with the general dietary guidelines for children in a single index. The index comprises 16 yes or no questions, including dietary habits that are in accordance with the principles of the Mediterranean dietary pattern and the general dietary guidelines for youth (e.g., consumption of at least one fruit at a daily basis, consumption of 2–3 fish per week, use of olive oil as the main culinary fat in salad and cooking, etc.), and other habits that undermine them (e.g., breakfast skipping, daily consumption of sweets, frequent consumption of fast food, etc.). Questions denoting a negative connotation with respect to a high-quality diet are assigned a value of −1, while those with a positive aspect are assigned a value of +1. Thus, the total KIDMED score ranges from −4 to 12 and is classified into three levels: ≥8, suggesting an optimal adherence to the MD; 4–7, suggesting an average adherence to the MD and an improvement needed to adjust dietary intake to guidelines; and ≤3, suggesting a low adherence to the MD and generally a low diet quality. With regard to physical activity habits, the questionnaire applied has been previously used in children in other large-scale epidemiological studies (13) and included simple closed-type questions regarding children’s frequency and time of participation in sports activities, active play, and sedentary activities. For the current analysis, student’s weekly frequency of participation in organized sports activities (range 0–7, i.e., from rare to daily participation) as well as weekly frequency (range 0–7, i.e., from rare to daily participation) and average duration (in hours) per bout of engaging in sedentary activities (such as television, computer and video games) were used. Daily time (in hours) spent in sedentary activities was also calculated for each student (*via* multiplying the weekly frequency of participation with the duration per bout of participation in sedentary activities and then dividing by 7). Using the threshold of 2 h/day proposed by current scientific evidence and guidelines (35–38), the students were classified as sedentary or not, i.e., exceeding (>2 h per day) or not (≤2 h per day) the recommended daily time spent in sedentary activities. All questionnaires were answered in Greek.

### Statistical Analysis

Data are presented as mean ± SD for continuous variables and as frequencies for categorical ones. Continuous variables were compared between groups using the Student’s *t*-test, and correlations between them were tested using the Pearson correlation coefficient (*r*), while the chi-square test was used to test differences between categorical variables. Multiple logistic regression analysis was used to explore the relationship between anthropometric or lifestyle parameters and the likelihood of total poor PF, with results presented as odds ratios (OR) with their corresponding 95% confidence intervals (95% CI) for each independent variable. The Hosmer–Lemeshow statistic was used to test models’ goodness-of-fit, and the Wald test was used to determine the hierarchy of independent variables regarding their contribution to the prediction of PF. All statistical analyses were performed using SPSS version 21.0 (SPSS Inc., IBM Hellas, USA). The significance level was set up at 0.05.

## Results

Students’ descriptive characteristics, in total and by gender, are presented in Table 1. In the whole study sample, 1.7% of the participating children were underweight, 21.9% were overweight, and 8.2% were obese. The prevalence of underweight was higher in girls compared to boys (2.0 versus 1.4%, *P* < 0.001), while more boys were overweight or obese compared to girls (22.2 versus 21.6% and 9.0 versus 7.5%, respectively, both *P* < 0.001). In addition, the prevalence of central obesity (WHtR ≥ 0.5) was 28.9% in the total study sample and was significantly higher in boys compared to girls (30.0 versus 27.7%, *P* < 0.001). Regarding lifestyle habits, only 39.8% of the participating children (boys: 39.6%, girls: 40.4%, *P* = 0.01) reported a high adherence to the MD (KIDMED score ≥ 8), the majority of the students enrolled (73.0%) participated in sports activities less frequently than three times/week (boys: 68.0%, girls: 78.3%, *P* < 0.001), while 23.1% of the study sample (boys: 25.5%, girls: 20.3%, *P* < 0.001) presented a high level of sedentariness (>2 h of engagement in sedentary activities per day).

**Table 1:** Descriptive characteristics of the participating children.

Characteristics	Total sample (*N* = 335,810)	Boys (*N* = 172,271)	Girls (*N* = 163,539)
Age, years	9.9 ± 2.8	9.9 ± 2.8	9.8 ± 2.8
Weight *z*-score		0.06 ± 1.03	−0.07 ± 0.96
BMI *z*-score		0.03 ± 1.03	−0.03 ± 0.97

Weight status (%)
Underweight	1.7	1.4	2.0
Normal weight	68.2	67.5	68.9
Overweight	21.9	22.2	21.6
Obesity	8.2	9.0	7.5
Waist to height ratio	0.48 ± 0.06	0.48 ± 0.06	0.47 ± 0.06
Central obesity (%)	28.9	30.0	27.7
KIDMED score (−4 to 12)	6.7 ± 2.4	6.7 ± 2.4	6.8 ± 2.3

**Adherence to the Mediterranean diet (%)**
High (KIDMED ≥ 8)	39.8	39.6	40.4
Moderate (KIDMED: 4–7)	50.5	50.3	50.5
Low (KIDMED ≤ 3)	9.7	10.1	9.1

**Participation in sports activities (%)**
High (>5 days a week)	8.6	10.4	6.7
Moderate (3–5 days a week)	18.4	21.5	15.1
Low (<3 days a week)	73.0	68.0	78.3

**Time spent in sedentary activities (%)**
Acceptable (≤2 h per day)	76.9	74.5	79.7
Increased (>2 h per day)	23.1	25.5	20.3

*Values are mean ± SD for continuous variables or frequencies for categorical ones*.

*BMI, body mass index*.

With regard to students’ performance in PF tests, 26.3, 48.3, and 25.4% were ≤25th, between the 25th and 75th and ≥75th percentile of normative age- and sex-specific values for the 20 m SRT test. Respective percentages for 10 × 5 m SRT were 17.5, 61.3, and 21.2%, for 30 s SUs 20.0, 56.7, and 23.3%, for SLJ 34.7, 43.5, and 21.8%, and for SR 26.4, 47.1, and 26.5%. When students’ performance in PF tests was stratified according to gender, boys performed better in the 10 × 5 m SRT test compared to girls, girls performed better in the SR test compared to boys, while performance in the 20 m SRT, 30 s SUs, and SLJ tests was similar between genders (data not shown). In all five PF tests, students’ performance, treated as absolute value, was negatively correlated with BMI, WC, WHtR, and weekly time spent in sedentary activities, and positively correlated with KIDMED score and frequency of weekly participation in sports activities (all *P* < 0.05, data not shown). In addition, students presenting low performance (≤25th percentile of published age- and sex-specific normative values) in PF tests had statistically significantly higher BMI, WC, and WHtR values, devoted more time in sedentary activities, whereas they adhered less to the MD and engaged less frequently in sports activities (all *P* < 0.05, data not shown).

**Figure 1:**
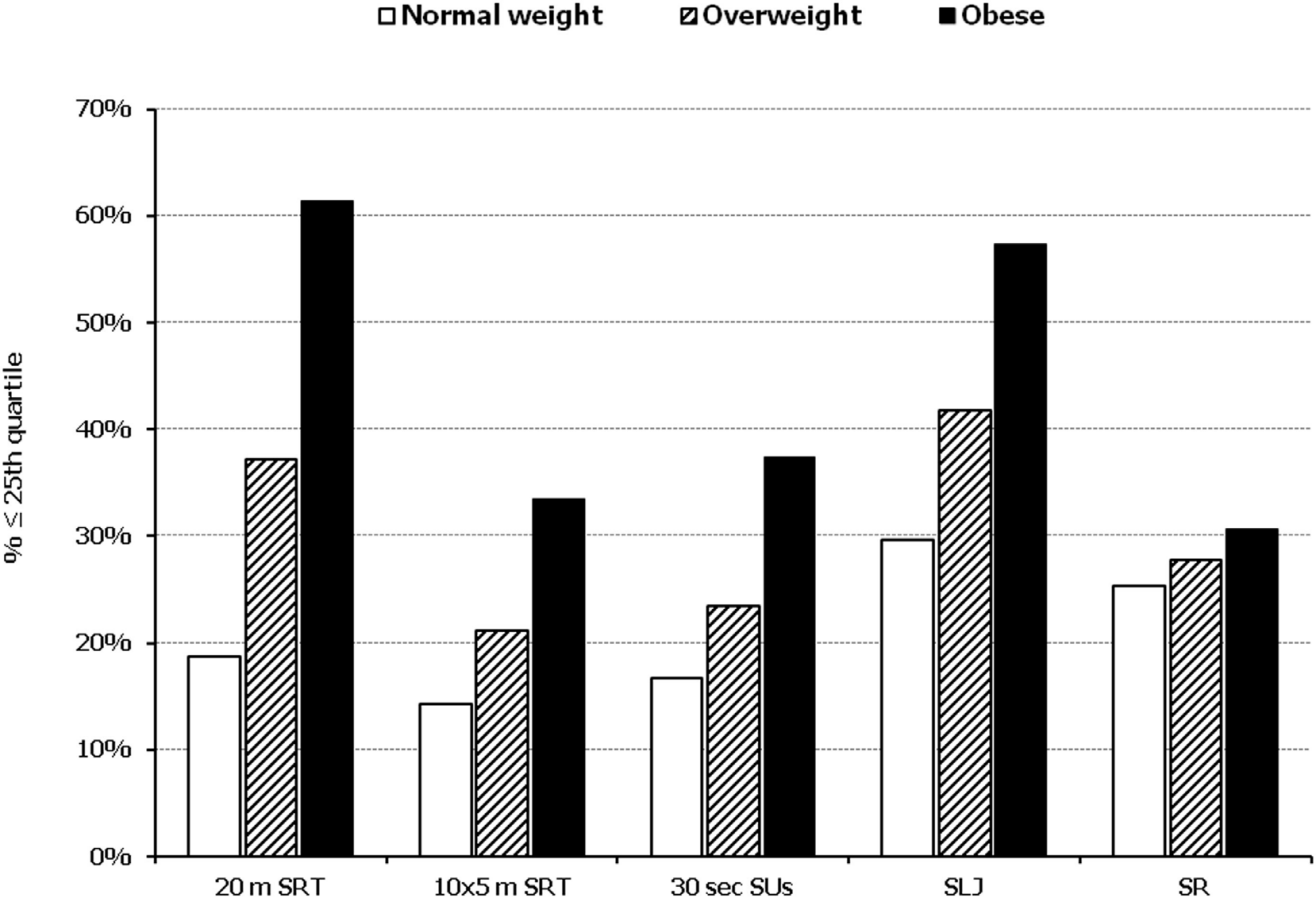
Illustration of the association between physical fitness level and weight status. Prevalence of low performance (<25th percentile of normative values) in 20 m shuttle run test (20 m SRT), 10 x 5 m shuttle run test (10 × 5 m SRT), sit-ups in 30 s (30 s SUs), standing long jump (SLJ), and sit and reach (SR) test, presented by student’s weight status (normal weight, overweight, and obese).

**Figure 2:**
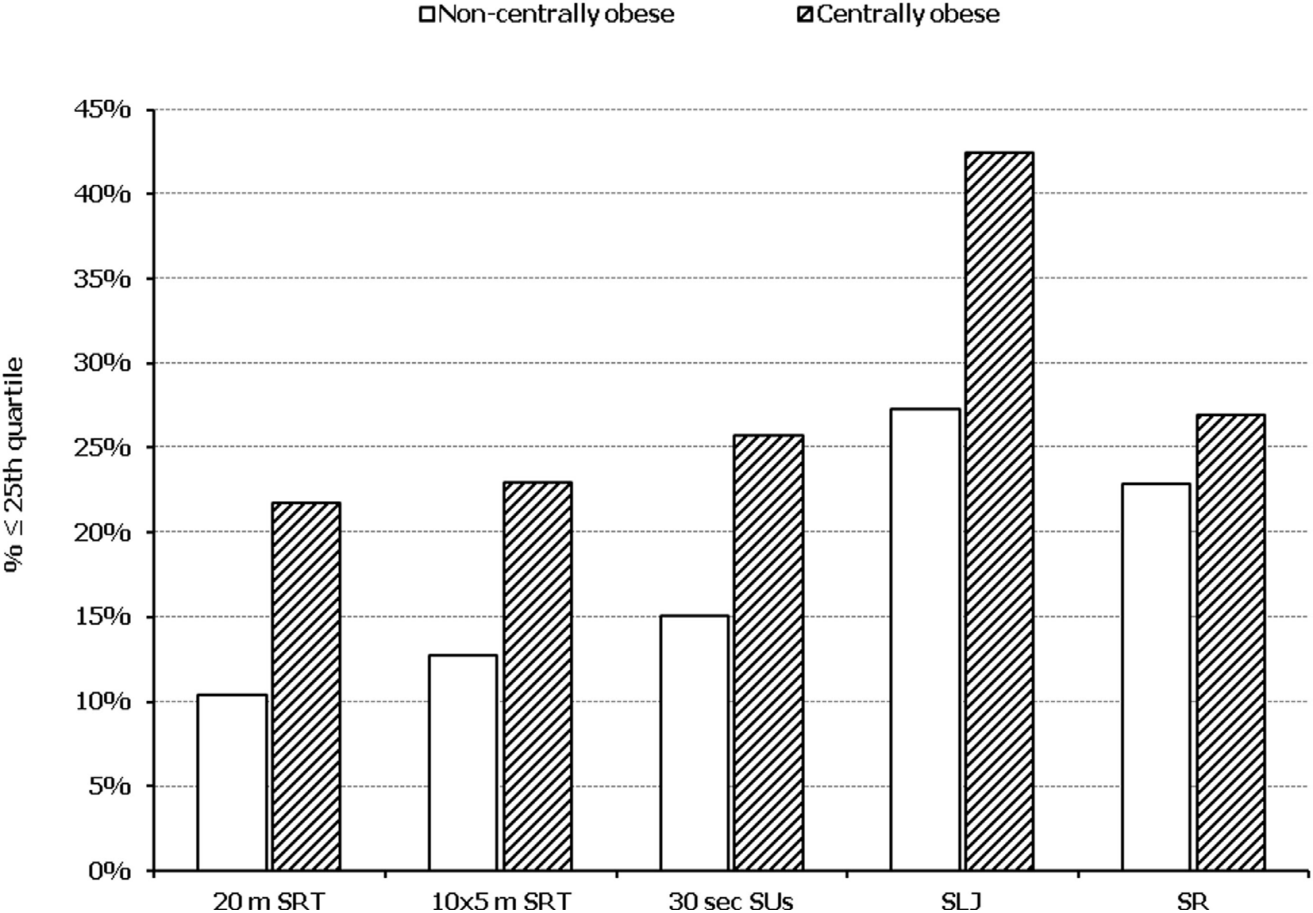
Illustration of the association between physical fitness level and presence of central obesity. Prevalence of low performance (<25th percentile of normative values) in 20 m shuttle run test (20 m SRT), 10 x 5 m shuttle run test (10 × 5 m SRT), sit-ups in 30 s (30 s SUs), standing long jump (SLJ), and sit and reach (SR) test, presented by student’s central obesity status (centrally obese and non-centrally obese).

**Figure 3:**
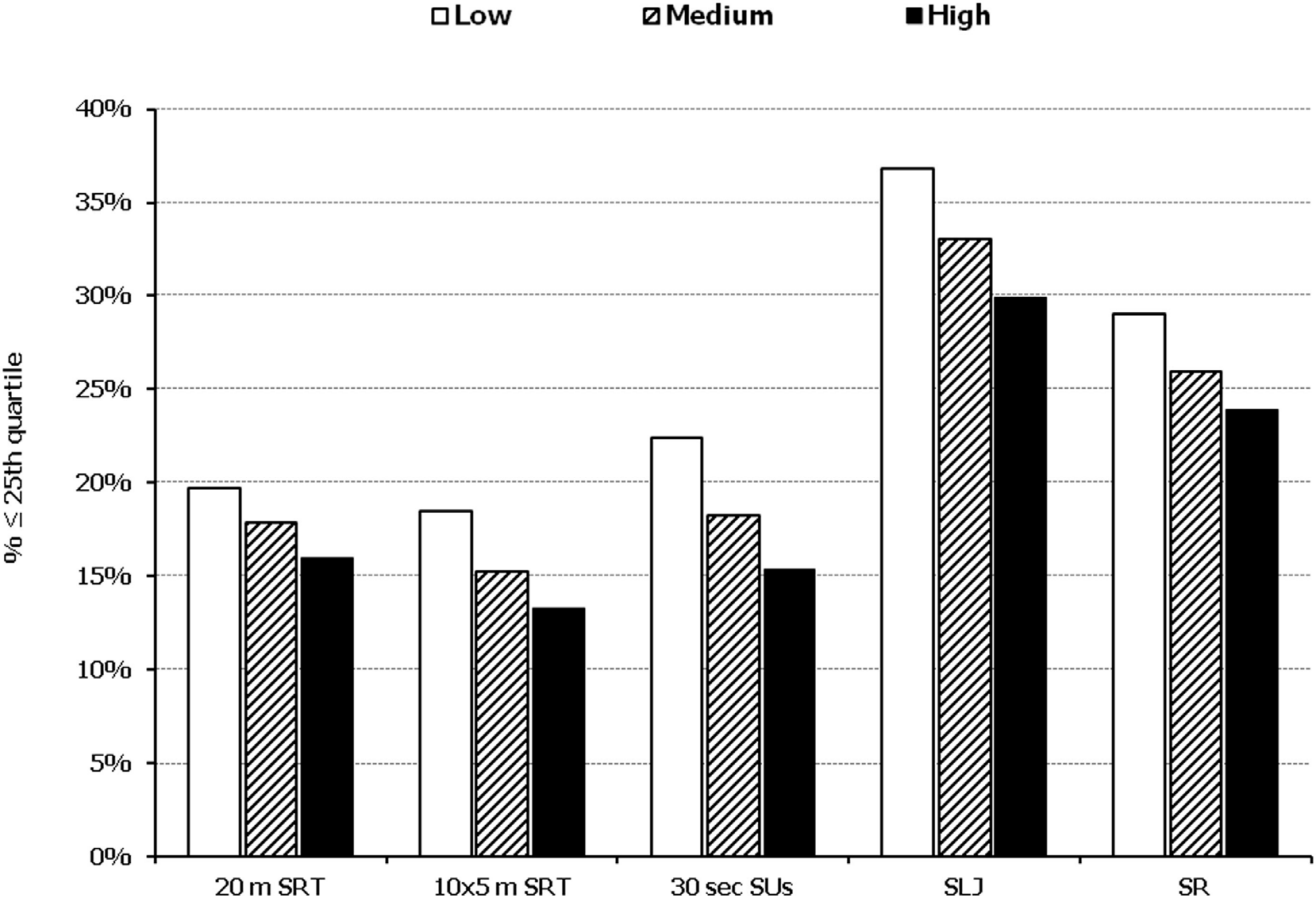
Illustration of the association between physical fitness level and adherence to the Mediterranean diet. Prevalence of low performance (<25th percentile of normative values) in 20 m shuttle run test (20 m SRT), 10 x 5 m shuttle run test (10 × 5 m SRT), sit-ups in 30 s (30 s SUs), standing long jump (SLJ), and sit and reach (SR) test, presented by student’s level of adherence to the Mediterranean diet (low, medium, and high).

**Figure 4:**
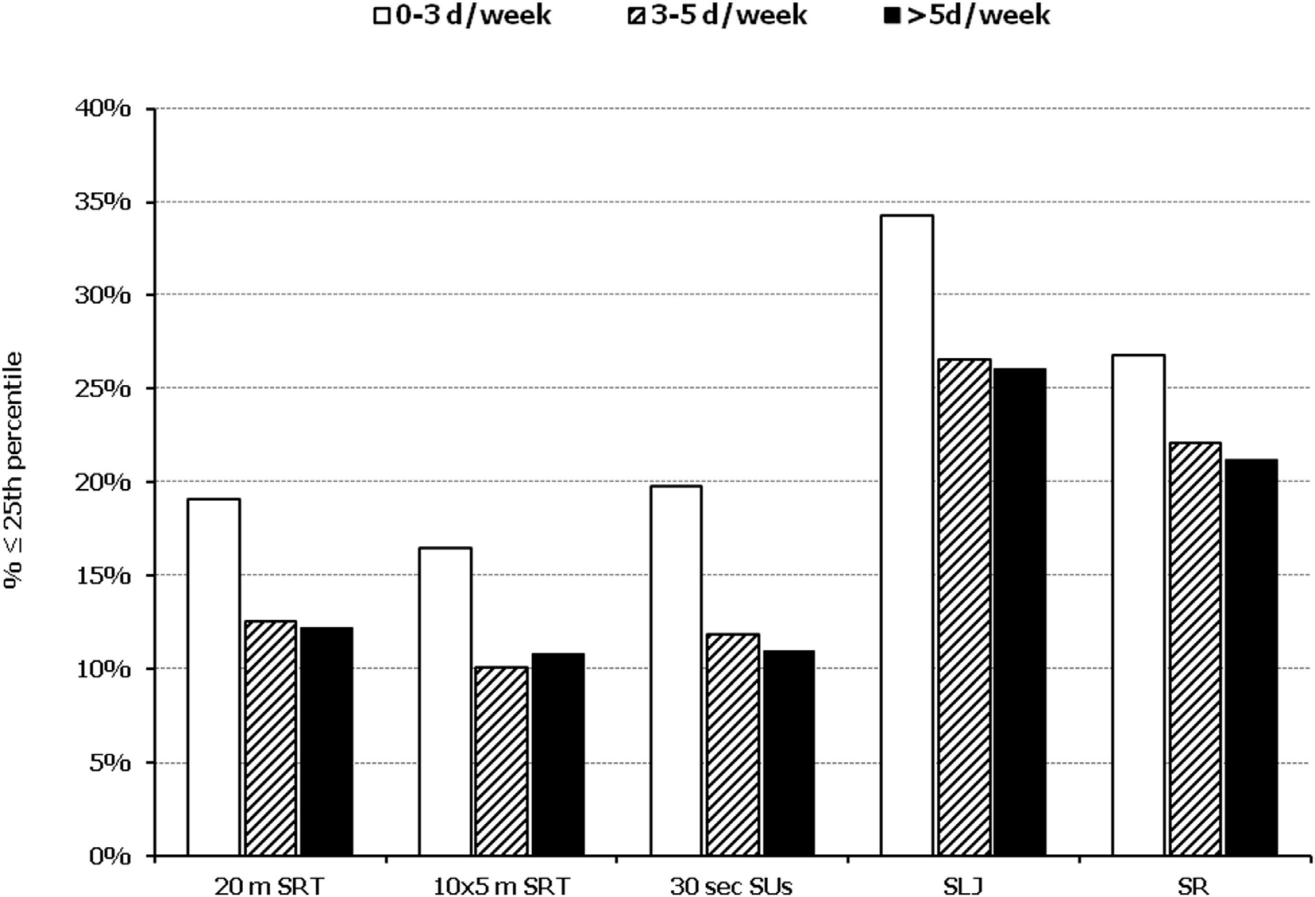
Illustration of the association between physical fitness level and weekly engagement to organized activities. Prevalence of low performance (<25th percentile of normative values) in 20 m shuttle run test (20 m SRT), 10 x 5 m shuttle run test (10 × 5 m SRT), sit-ups in 30 s (30 s SUs), standing long jump (SLJ), and sit and reach (SR) test, presented by student’s weekly engagement to organized sports activities (0–3, 3–5, and >5 days a week).

**Figure 5:**
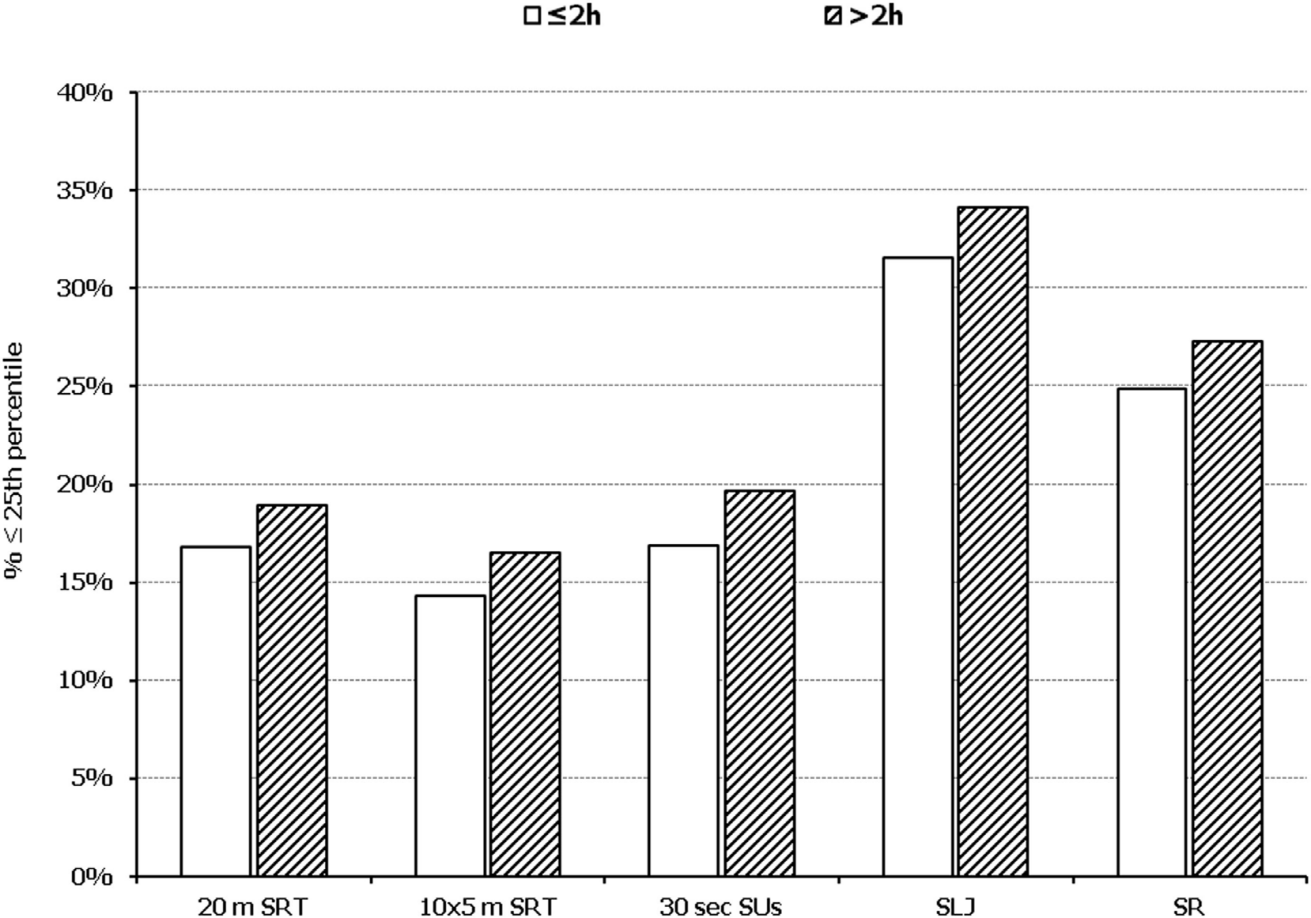
Illustration of the association between physical fitness level and time spent in sedentary activities. Prevalence of low performance (<25th percentile of normative values) in 20 m shuttle run test (20 m SRT), 10 x 5 m shuttle run test (10 × 5 m SRT), sit-ups in 30 s (30 s SUs), standing long jump (SLJ), and sit and reach (SR) test, presented by student’s time spent in sedentary activities (≤2 and >2 h per day).

In all five PF tests applied, the performance of students was negatively associated with increased adiposity. Indicatively, the percentages of normal-weight, overweight, and obese students ≤25^th^ percentile, based on published norms, were as follows: 18.7, 37.2, and 61.4% for 20 m SRT, 14.3, 21.2, and 33.5% for 10 × 5 m SRT, 16.7, 23.5, and 37.4% for 30 s SUs, 29.6, 41.8, and 57.3% for SLJ, and 25.3, 27.8, and 30.6%, for SR, respectively (Figure 1). Likewise, the performance of students was negatively associated with the presence of central obesity. Specifically, the percentages of non-centrally obese and centrally obese students ≤25th percentile for PF tests were as follows: 10.4 versus 23.8% for 20 m SRT, 12.7 versus 23.0% for 10 × 5 m SRT, 15.1 versus 25.8% for 30 s SUs, 27.3 versus 42.5% for SLJ, and 22.9 versus 27.0% for SR, respectively (Figure 2). With regard to student lifestyle habits, PF performance had a positive association with level of adherence to the MD (Figure 3), a positive association with the rate of weekly participation in organized sports activities (Figure 4), and a negative association with daily time spent in sedentary activities (Figure 5). According to multiple logistic regression analysis (Table 2, Model 1), the presence of overweight/obesity (OR: 4.43, 95% CI: 3.98–4.93), low adherence to the MD (KIDMED ≤ 3) (OR: 1.27, 95% CI: 1.09–1.48), and increased time spent in sedentary activities (>2 h per day) (OR: 1.16, 95% CI: 1.03–1.29) were positively associated with poor PF, after adjusting for age and sex, whereas for every 1 day increase in the weekly frequency of engagement in athletic activity, the probability of poor PF decreased by 26% (OR: 0.74, 95% CI: 0.72–0.77). In a similar model incorporating central instead of general adiposity, the presence of WHtR ≥ 0.5, indicative of abdominal adiposity, emerged as an even stronger possible predictor of total poor PF (OR: 5.19, 95% CI: 4.66–5.78, *P* < 0.001), after adjusting for the same factors (Table 2, Model 2).

**Table 2:** Multiple logistic regression analysis, evaluating the association between anthropometric characteristics (model 1: presence of overweight or obesity, model 2: presence of central obesity) and lifestyle habits and the probability of low total physical fitness^a^.

	Wald	OR	95% CI
	**Model 1^b^**		

Overweight/obesity	751.1	4.43	3.98–4.93
Low adherence to the Mediterranean diet (KIDMED score ≤ 3)	9.3	1.27	1.09–1.48
Increased time spent in sedentary activities (>2 h per day)	6.2	1.16	1.03–1.29
Weekly engagement in physical activity (days per week)	353.9	0.74	0.72–0.77

	**Model 2^b^**		

Central obesity (WHtR ≥ 0.5)	892.2	5.20	4.66–5.78
Low adherence to the Mediterranean diet (KIDMED score ≤ 3)	8.1	1.25	1.07–1.46
Increased time spent in sedentary activities (>2 h per day)	5.9	1.15	1.03–1.29
Weekly engagement in physical activity (days per week)	310.4	0.76	0.73–0.78

*^a^Defined as the concomitant performance ≤ 25th percentile of published normative values in 20 m shuttle run test, 10 x 5 m shuttle run test, sit-ups in 30 s, standing long jump, and sit and reach test*.

*^b^Adjusted for age and sex*.

*OR, odds ratios; CI, confidence interval; WHtR, waist to height ratio*.

## Discussion

In the present study, we evaluated PF levels in relationship with anthropometric characteristics and lifestyle habits and assessed the most significant determinants of low PF levels in a nationally representative sample of Greek students. The major findings from our data are that the presence of obesity and central obesity are both negatively associated with all the components of PF that we examined and are the most aggravating factors responsible for total low PF, accompanied by lack of engagement in physical activity, poor dietary habits, and sedentariness.

Overweight and obesity have been repeatedly identified as the cornerstone for numerous health pathologies during childhood (15–17). In our study, excess body weight was associated with poor PF, a fact that is in accordance with other studies supporting that overweight or obese children are not sufficiently active and consequently appear to be less physically fit. The most eminent explanation is that weight-bearing exercises, i.e., exercises that require “carrying” someone’s body mass, are disadvantageous for children with excess body weight (39, 40). Noteworthy, according to our results, the presence of central obesity was an even stronger predictor of poor PF in childhood than general adiposity. Although the association between abdominal obesity and PF has not been thoroughly examined in the literature, it is well-documented that the prevalence of central obesity is nowadays particularly high among children, and despite the attention given to the epidemic of childhood obesity, WC has increased at a higher rate than total body weight over the past 10–30 years in the youth (13). In addition, the presence of central obesity in children has been proposed to have a higher predictive value with regard to metabolic disorders and chronic diseases, compared to whole-body fat mass, a fact that suggests a parallel increase in the cardiometabolic risk of children and adolescents worldwide (29, 41). Besides its well-established metabolic and inflammatory complications (42), this negative impact of central obesity on cardiometabolic indices could also be partially explained by its negative association with PF levels observed in the present study, a fact that needs further investigation. Based on the aforementioned, the use of WHtR or other indices to assess abdominal adiposity in the routine pediatric practice should be encouraged, and children presenting with central obesity should undergo a further assessment in terms of lifestyle, PF, and cardiometabolic risk.

Besides adiposity, the second most important predictor of PF concluded by the results in our study was the frequency of engagement in physical activity, a reasonable observation given that the relationship between increased physical activity and ameliorated PF is well documented (7, 43, 44). However, children nowadays are more sedentary than ever. Reasons for that are multiple and include the widespread use of computer and video games along with increased time of television viewing, inactive role models (e.g., parents), limited free time, unsafe environment, lack of sport facilities or insufficient funds to participate in certain recreation programs, and insufficient access to quality daily physical education (22–24). In contrast, regular physical activity (regardless of its type; strength or aerobic exercise) is essential for weight regulation and/or reduction, increases in bone health and muscle strength, and improvements in cardiovascular function (45). At the same time, it is beneficial psychologically, since it is associated with an increase in self-esteem and self-concept and a concomitant decrease in anxiety and depression (45). Given the aforementioned, along with the finding that the higher the participation in organized sports the lower the presence of children with low PF, it can be reasonably suggested that the lesson of physical education in schools can serve as an important tool for promoting student engagement in athletic activities and reducing sedentariness, and presumably, in the long term, as a way of improving their PF level.

An interesting finding of our study is that the level of adherence to the MD was also a significant predictor of PF level, following adiposity and engagement in physical activity. According to accumulated epidemiological and interventional studies, the adoption of a healthy dietary pattern such as the MD has been proven to be efficient not only in protecting against the development of but also in the management of several chronic diseases, including cardiovascular diseases, diabetes mellitus, neurodegenerative diseases, and obesity (46). Regarding its association with PF, direct data are scarce; however, it is well established that nutrition is an important part of athletic performance especially during childhood and adolescence, in addition to allowing for optimal growth and development (47). Furthermore, published data show a positive relationship between the degree of adherence to the MD and physical activity (43, 48, 49), as well as a negative relationship with indices of abdominal adiposity (50) in children. Thus, it can be proposed that such a model of a well-balanced diet, providing adequate energy sources throughout the day, rich in all essential nutrients and natural antioxidants, poor in saturated fat, and based on an abundant consumption of fruits, vegetables, legumes, fish, nuts, and olive oil, can also improve PF during childhood, especially when combined with increased physical activity and given that it is associated with a more favorable body composition. Therefore, besides the promotion of a physically active lifestyle, strategies aiming at improving schoolchildren’s dietary habits toward a prudent dietary pattern, such as the MD, are also crucial, as a mean to improve their PF and safeguard their health in general.

A limitation of our study derives from its design. Cross-sectional studies are generally limited in providing causal relationships and can only generate hypotheses about the possible links between parameters, in this case between anthropometric and lifestyle characteristics and poor PF during childhood and adolescence. Although our data provide evidence that anthropometric and lifestyle habits are closely associated with PF in childhood, it should be noted that several other parameters could also have an impact on PF, indirectly through an effect on lifestyle habits. Nevertheless, factors such as family socioeconomic status, parental educational level, and residential environment characteristics (e.g., access to green spaces or athletic facilities) were not assessed in the present study, mainly due to practicality reasons. Moreover, although a clear methodology was implemented in order to ensure accuracy in student anthropometric and lifestyle assessment, under- or overestimations cannot be excluded. This limitation is more evident with regard to the lifestyle questionnaire used, which may have been difficult for younger students to answer. However, we tried to overcome this restriction through implementing a standardized protocol, according to which students provided their answers with the assistance of their previously well-trained teachers. Last but not least, it should be mentioned that several additional measurements, which were not economically feasible due to the large sample size, could add to the accuracy and strength of our results, including a more comprehensive assessment of PF using all nine Eurofit tests, as well as the assessment of body composition for a more accurate estimation of adiposity.

In conclusion, anthropometric characteristics such as excess body weight and increased abdominal fat accumulation, as well as lifestyle parameters including the lack of physical activity and the adoption of poor dietary habits, are strongly and positively associated with poor PF in childhood and adolescence. It is urgent that large-scale interventions and relevant public health policies are designed and implemented toward the promotion of PF level of the pediatric population and ultimately their health status. In the context of such interventions and health policies, cardiorespiratory fitness should be the primary target, given that it is the most well-studied component of PF, with the majority of studies emphasizing on its significant preventative and health-promoting role both in children and adults (6, 7, 44). Our results suggest that emphasis should be placed primarily toward preventing childhood obesity and abdominal adiposity and secondarily toward providing schoolchildren with the adequate stimuli for engaging in physical activity and following a prudent dietary pattern, with a view to accomplish beneficial changes in their PF and health in general.

## Ethics Statement

The study was conducted according to the guidelines laid down in the Declaration of Helsinki, and all procedures involving human subjects/patients were approved by the Review Board of the Ministry of Education and the Ethical Committee of Harokopio University of Athens.

## Author Contributions

DP, KT, GA, and LS contributed to the study concept and design and critically revised the manuscript; GP, AM, DK, EB, and MG contributed to the acquisition, analysis, and interpretation of the data and performed the statistical analyses; and GA wrote the first draft of the manuscript. The results of the present study are presented clearly, honestly, and without fabrication, falsification, or inappropriate data manipulation.

## Conflict of Interest Statement

The authors declare that the research was conducted in the absence of any commercial or financial relationships that could be construed as a potential conflict of interest.
